# Transcending Traditional Treatment: The Therapeutical Potential of Nanovesicles for Transdermal Baclofen Delivery in Repeated Traumatic Brain Injury

**DOI:** 10.34172/apb.2024.031

**Published:** 2024-03-09

**Authors:** Nermin M. Sheta, Amira A. El-Gazar, Ghada M. Ragab, Marwa A. Essa, Khaled M. Abdel-Haleem, Rania Moataz El-Dahmy

**Affiliations:** ^1^Pharmaceutics Department, Faculty of Pharmacy, October 6 University, Giza, Egypt.; ^2^Pharmacology and Toxicology Department, Faculty of Pharmacy, October 6 University, Giza, Egypt.; ^3^Pharmacology and Toxicology Department, Faculty of Pharmacy, Misr University for Science & Technology (MUST), Giza, Egypt.; ^4^Biochemistry Department, Faculty of Pharmacy, October 6 University, Giza, Egypt.

**Keywords:** Baclofen, FAK, GABAB, Nanoparticles, Transdermal, Traumatic brain injury

## Abstract

**Purpose:**

The repositioning of previously approved drugs is occupying the researchers’ plans. Baclofen (Bac) was our candidate for its established neuroprotective capacity, with a proposal of efficient drug delivery as non-ionic surfactant-based nanovesicles (NISNV) formulae against mild repetitive traumatic brain injury (mRTBI) in rats, thus reducing the number of orally or injected medications, especially in severely comatose patients or pediatrics.

**Methods:**

A (2^3^) factorial design was implemented for confining Bac-loaded NISNV formulae, where a bunch of variables were inspected. An *in-vivo* experiment was done to test the prepared formula’s efficacy transdermally. The following parameters were measured: brain expression of gamma amino butyric acid _B_ (GABA_B_), protein kinase C- α (PKC-α), focal adhesion kinase (FAK), TNF-α and nuclear factor kappa B (NF-κB) p65, malondialdehyde (MDA), superoxide dismutase (SOD), and histopathology.

**Results:**

The particle size (PS) and entrapment efficiency percent (EE%) speckled from 60.40±0.28% to 88.02±0.01% for the former and 174.64±0.93 to 1174.50±3.54 nm for the latter. *In vitro* release% after 8 hours ranged from 63.25±5.47% to 84.79±3.75%. The optimized formula (F4) illustrated desirability=1, with 630.09±3.53 µg/cm^2^ of Bac permeated over 8 hours, which equates to 100% of Bac. Bac post-trauma treatment restored brain expression of GABA_B_ and PKC-α, while decreasing FAK. Besides enhancing the histological findings, the anti-inflammatory effect was clear by decreasing TNF-α and NF-κB p65. Consequently, significant antioxidant sequelae were revealed herein by diminishing MDA levels and restoring SOD activity.

**Conclusion:**

Transdermal delivery of Bac-loaded niosomes confirmed neuroprotection and succeeded in surpassing skin-to-brain barriers, which makes it a promising therapeutic option for repeated traumas.

## Introduction

 There is an increasing global awareness to the consequences of mild and recurrent brain injury, especially in athletes and military personnel, whereas the primary injury cannot be prevented.^1^ It was estimated worldwide that from 64 to 74 million traumatic brain injury (TBI) cases may occur each year.^2^ The region of the brain that contacts the skull can suffer damage following a forceful hit to the head as the brain collides with the intracranial surface of the skull. Furthermore, any rotational movement to the brain after this collision may cause “diffuse axonal injury”, which was suggested to cause neuronal degeneration.^3^ Patients with TBI experience cycles of neuropsychiatric and physiological abnormalities regardless of severity, but it depends on the individual’s vulnerability to cope with the consequences of secondary injury. There is a scarcity of prospective findings limiting the return to the normal routine activity of TBI patients, such as in athletes or highly susceptible persons. Thus, there is an urgent demand for new treatment strategies or adaptive reuse/relocation of medication to diminish the consequences of neurodegeneration with TBI, especially repetitive ones (RTBI).^4,5^

 Baclofen (Bac), a γ-aminobutyric acid (GABA) agonist, works by activating GABA_B_ receptors, which are mostly connected to G-protein-coupled receptors. This causes hyperpolarization, which lowers excitotoxicity and the rate at which neurons fire.^6,7^ The drop in firing could also be due to the presynaptic inhibitory regulation of glutamatergic transmission through GABA_B_ heteroreceptors, which are found at glutamatergic terminals. Moreover, Bac blocks calcium influx by limiting glutamate release before it reaches the synapses.^6,8^

 The pharmacological support for Bac has been a topic of debate due to contradictory findings that may be related to interruptions in the oral administration of the drug. Oral administration of Bac is difficult for patients with swallowing difficulties or high-dose requirements. In addition, it causes many gastrointestinal side effects and requires administering the drug multiple times a day, owing to baclofen’s short half-life. Moreover, after taking Bac orally, it is extensively metabolized by the liver, reducing its therapeutic efficacy.^9^

 Hence, there is a need to develop an alternative delivery method like the Bac transdermal system, which aims to improve therapeutic outcomes by bypassing the first-pass effect of Bac, avoiding alimentary side effects, and taking advantage of their lipophilic nature to offer sustained drug release, providing more consistent Bac levels.^10^

 Unfortunately, the transdermal drug delivery system faces a big difficulty with the stratum corneum of the epidermis. This difficulty could be overcome by non-ionic surfactant-based nanovesicles (NISNV). NISNV is comprised of a bilayer of non-ionic surfactants, which could enhance drug penetration through the skin.^11^ The augmented potential of NISNV to facilitate permeability might be attributed to the transfer of drugs across the membrane through alteration of the lipid pattern, affecting stratum corneum function via increasing its hydration and hence loosening the tightly packed cellular structure.^12^

 Moreover, NISNV could encapsulate hydrophilic and lipophilic drugs, permitting them to be employed in the delivery of a wide range of drugs. NISNV can also enhance the solubility and biological efficacy of poorly water-soluble drugs such as Bac.^13^ NISNV have gained significant attention as a drug delivery system due to their safety, biocompatibility, low toxicity compared to other surfactants, biodegradability, and modifiability to enhance their stability and prolong drug release, allowing for versatile and targeted drug delivery on a large scale.^14-16^

 Therefore, this study aimed to examine the influence of Bac as a regulator of the GABA_B_ receptor in the context of RTBI. We sought to achieve this by creating and assessing a transdermal Bac-loaded NISNV, which can increase Bac solubility and permeability through the skin and offer enhanced therapeutic results while minimizing the potential for negative side effects. In comparison to oral medication, the transdermal approach presents a safer and more effective option for treating RTBI patients.

## Materials

 Baclofen was received as a sample from Misr Pharmaceutical Industries Company, Abu Zaabal, Egypt; Baclofen Tablets (Bac tab) B.P. 2011 10 mg, 20 mg (MISR, Al DELTA PHARMACEUTICALS TRADE CO.); Tween 80 and 20 (T80, T20) (Merck, Germany); Cholesterol (Acros Organics, New Jersey, USA); Methanol and Triethanolamine (TEA) (Adwic, El-Nasr Pharmaceutical Chemical Company, Egypt); Carbopol 940 (BF Good Rich Company, Ohio, USA); Newborn rat skin (the animal house of the applied research center for medicinal plant ARCMP, Egypt).

###  The study plan

 The proposed strategy outlined in Table 1 includes examining certain preparatory factors, such as the impact of different types of surfactant (SAA), T80, and T20, varying the surfactant/cholesterol ratio (SAA/CH) at 1:1 and 4:1, and altering sonication time (0 and 3 minutes) to assess their influence on particle size (PS) and entrapment efficiency (EE).

**Table 1:** 2^3^ full factorial experimental design to optimize Bac-loaded NISNV formulae

**Factors**	**Levels**	
Surfactant type	Tween 80	Tween 20
Surfactant: Cholesterol ratio	1:1	4:1
Sonication time	0	3
**Responses**	**Desirable constraints**	
EE (%)	Maximize	
PS (nm)	Minimize	

EE: Entrapment efficiency; PS: Particle size.

###  Preparation of baclofen NISNV

 Bac-loaded NISNV was achieved through the conventional thin-film hydration technique. Table 2 depicts the composition of Bac NISNV formulae. In a round-bottom flask, 10 mL of methanol was placed, and then SAA, Bac, and CH were precisely weighed and dissolved. The mixture was evaporated under reduced pressure at 65 °C and rotated at 150 rpm via a rotary evaporator (Heidolph, Germany) till a thin, dried film emerged on the flask’s inner wall. This film was then hydrated with 10 ml of distilled water using a rotary evaporator under normal pressure. Then the formula was permitted overnight at 4 °C.^17^

**Table 2:** Experimental design, variables, and response outcomes of 2^3^ factorial designs of Bac-loaded NISNV formulae

**Formula code**	**X**_1_ **Surfactant type**	**X**_2_ **Surfactant/Cholesterol ratio**	**X**_3_ **Sonication time (min)**	**Y**_1_ **EE (%)**	**Y**_2_ **PS (nm)**
F1	T80	1:1	0	80.42 ± 0.30	1174.50 ± 3.54
F2	T80	1:1	3	66.10 ± 0.07	576.40 ± 0.57
F3	T80	4:1	0	88.020 ± 0.01	420.30 ± 0.42
F4	T80	4:1	3	85.20 ± 0.47	174.64 ± 0.93
F5	T20	1:1	0	73.225 ± 0.16	806.99 ± 0.01
F6	T20	1:1	3	60.40 ± 0.28	414.10 ± 0.14
F7	T20	4:1	0	81.13 ± 0.07	257.51 ± 0.40
F8	T20	4:1	3	73.17 ± 0.83	208.20 ± 1.13

All formulae contain 10 mg Bac. Data are presented as average mean ± SD, n = 3. EE: entrapment efficiency; PS: particle size.

###  Evaluation of the various formulated Bac formulae

####  Determination of drug entrapment EE% using the cooling centrifugation method

 EE% was calculated indirectly by estimating the amount of free Bac that remained dissolved in the suspension medium after cooling centrifugation (Union 32R, Hanil Science Industrial CO., Korea). The cooling centrifuge is a specialized centrifuge that incorporates a cooling system to maintain low temperatures during the centrifugation process, where the low temperature applied preserves the integrity and functionality of the nanovesicles during the centrifugation steps.^18^ One mililiter of each formula was centrifuged at 14 000 rpm at -4 °C for 1 hour. The vesicles were separated from the supernatant, washed twice with methanol, and re-centrifuged. Thereafter, the concentration of free Bac was estimated spectrophotometrically (Shimadzu (2401/PC), Japan) at 266.8 nm, and the EE% was calculated as follows^17,19^:


$$EE\%=\frac{Total\;amount\;of\;drug-amount\;of\;unbound\;drug}{Total\;amount\;of\;drug}*100$$

###  Particle size (PS) and zeta potential measurement (ZP)

 As mentioned in Appendix 1.^20-23^

###  In-vitro release of Bac-loaded NISNV

 As mentioned in Appendix 2. ^24-27^

###  Evaluation tests of the optimized Bac NISNV formula

 The optimized Bac NISNV formula (F4) was decided based on the desirability value computed using Design-Expert® software and the release outcomes based on the ANOVA statistical outcomes, achieving the highest EE% and lowest PS.

###  Ex-vivo permeation of Bac NISNV optimum formula through excised newborn rat skin

*Ex-vivo* drug permeation was carried out through newborn rat skin over 8 h in 1% w/v T80 in PB solution (pH = 7.4) at 37 ± 0.5 ºC for the optimized (F4) Bac-loaded NISNV gel and the control Bac gel as control. The optimized formula F4 and its Bac suspension were converted into a gel by adding 1% (w/w) Carbopol 940 gel and a sample from Bac NISNV gel (containing 2 mg Bac) and its Bac suspension gel (Bac gel) were spread over the newborn rat skin held on a 2.5 cm diameter glass cylinder tube with the aid of a waterproof plaster in a dissolution vessel containing 100 mL 1% w/v T_80_ in PB (pH = 7.4)^28^ for 8 h at 37 ± 0.5 ºC, and stirring speed equals to 100 rpm. Samples (5 mL) were rescinded every hour, followed by replacement with the same volume of fresh medium. The concentration of Bac was analyzed at 268 nm. The cumulative quantities of permeated Bac *via* the skin per unit surface area were calculated and used for plotting the profiles of drug permeation per unit time (t).

###  Fourier transform infrared spectroscopy (FT-IR) and X-Ray powder diffraction (XRD)

 As mentioned in Appendix 3.^29-31^

###  Transmission electron microscopy (TEM) surface morphology

 The morphology of F4 was mounted on a film-coated copper grid that had been dyed with a drop of 2% phosphotungstic acid, then left for drying, followed by examination using TEM (JEOL, JEM-1230, Japan).^17,19^

###  Pharmacological investigation outcomes

####  Selecting the effective dose of oral baclofen tablets (Bac tab) in the mild repetitive traumatic brain injury (mRTBI) model

 Since Sprague-Dawley are the most frequently used rat strains in modelling TBI,^32^ healthy adult Sprague-Dawley rats (250-300 g) were acquired from the animal house of the National Research Center, Giza, Egypt. Before the experimental procedures, animals were given a week to familiarize themselves with their surroundings while having free access to tap water and pelleted standard rat food. The protocol followed the Guidelines for the Care and Use of Laboratory Animals’ animal welfare requirements (NIH publication, 1996). The previously discussed approach^33,34^ was used to direct the induction of repetitive trauma, and it was approved by the Research Ethical Committee of the Faculty of Pharmacy, October 6 University (Giza, Egypt), Number: PRE-Ph-2202014.

 Using 20 male adult rats, a dosage range-finding study was conducted, guided by a previous study of baclofen as a GABA_B_ receptor modulator,^35^ to determine the optimum neuroprotective dose of Bac tab_10_ or_20_ (10 or 20 mg/kg). Briefly, in this part, rats were anesthetized by inhaling isoflurane (4%), which was preserved through vaporization at a concentration of 1.5% throughout the experiment. Following that, the right anterior frontal area (1.5 mm lateral to the midline in the mid-coronal plane) was chosen as the site where the weight (75 g) was released from a height of 25 cm to produce a 0.5-joule final impact. All animals, except for negative control ones, were exposed to this technique once daily for a total of five successive days to produce mRTBI. Then, traumatized animals were blindly assigned into three groups: (mRTBI) group: rats were left without any further intervention for 7 successive days; (mRTBI + Bac tab_10_) and (mRTBI + Bac tab_20_) groups: rats were post-treated orally by Bac tab (10 mg/kg and 20 mg/kg, respectively) for 7 successive days.

###  Identification of neuroprotective capacity of Bac tab_20_ and transdermal formula against mRTBI

 In this part of the study, 40 male rats were used and distributed over 5 groups (n = 8).

- Group I (control): Normal rats received distilled water daily until the end of the study.
- Group II (mRTBI): Rats were traumatized on their heads once per day for 5 days, as previously described in the dose selection part.
- Group III (mRTBI + Bac tab_20_): Rats were traumatized as in Group II, then treated immediately after the last trauma with Bac tab_20_ (20 mg/kg, once/day, orally).
- Group IV (mRTBI + Bac gel): Rats were traumatized as in Group II, then treated immediately after the last trauma with Bac gel (20 mg/kg, once /day, transdermally, for 7 days).
- Group V (mRTBI + F4): Rats were traumatized as in Group II, then treated immediately after the last trauma with F4 (20 mg/kg, once /day, transdermally, for 7 days).

Notably, all the animals’ backs, even the control group, were shaved before the last trauma to neutralize all the circumstances, and Bac gel and F4 were topically applied to cover the shaved back surface.

###  Collection of samples

 As mentioned in Appendix 4.

###  Biochemical measurements

 As mentioned in Appendix 5.^36^

###  Histopathological investigations

 As mentioned in Appendix 6.^36^

###  Statistical analysis

 The data are shown as mean ± SD (n = 8). One-way ANOVA was applied to perform multiple comparisons, and then Tukey Kramer was used as a post hoc analysis with the aid of the statistical analysis and graphing program GraphPad Prism (ISI^®^, San Diego, CA, USA) (version 5).

## Results and Discussion

###  Evaluation of the differently prepared Bac-NISNV formulae

####  Determination of drug encapsulation efficiency percent (EE%) via the cooling centrifugation method

**Figure 1:**
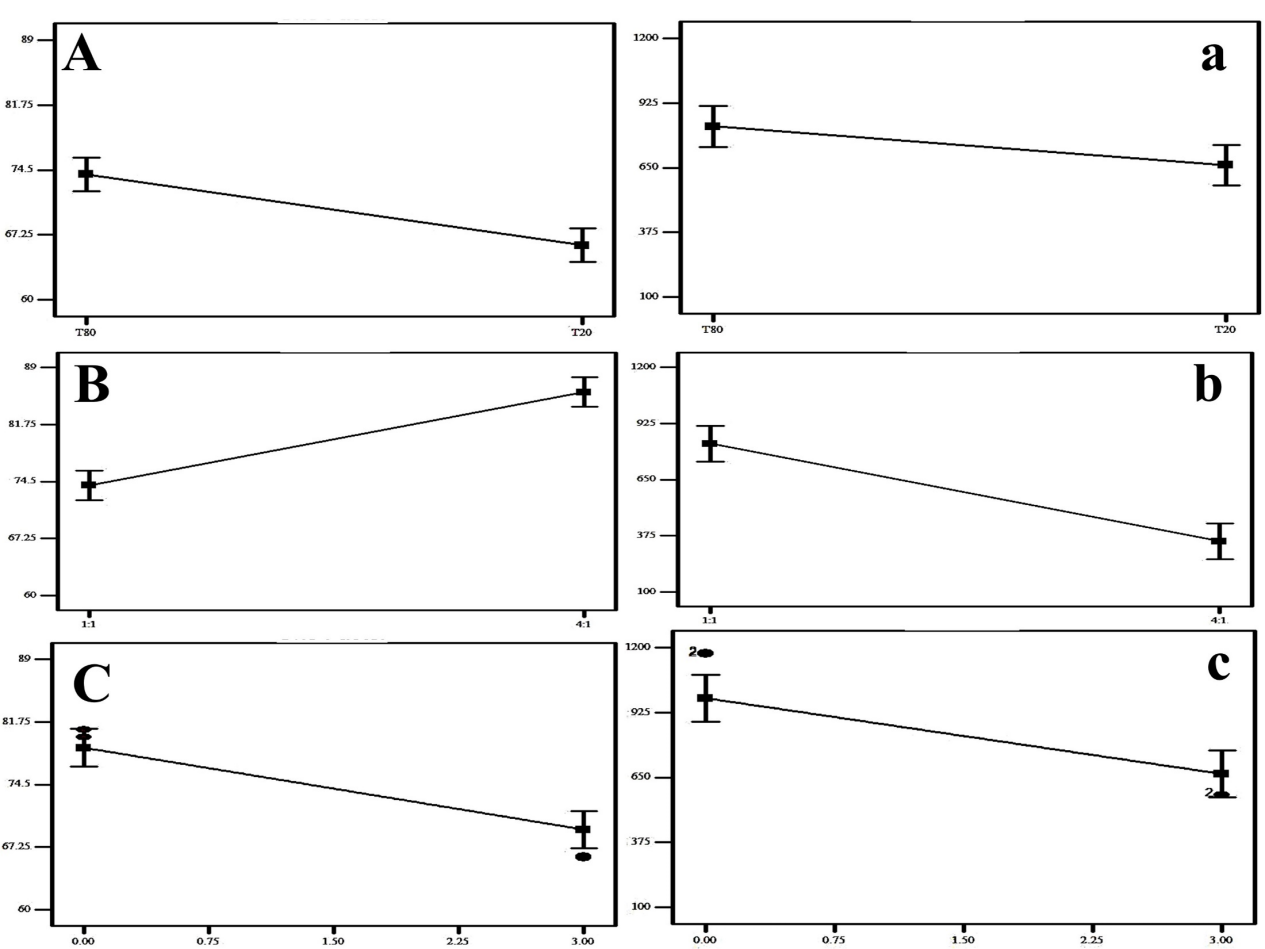
The impact of surfactant type (A), surfactant: cholesterol ratio (B) and sonication time (C) on EE % and the impact of surfactant type (a), surfactant: cholesterol ratio (b) and sonication time (c) on PS (n = 3)

 The *EE%* of the differently prepared Bac NISNV formulae varied from 60.40 ± 0.28% to 88.02 ± 0.01%, as shown in Table 2 and illustrated in Figure 1. Regarding the effect of SAA type (X_1_), it was observed that the EE% of NISNV prepared *via* T_80_ (80.42 ± 0.30% for F1) was significantly greater (*P* < 0.0001) in contrast to that prepared using T_20_ (73.225 ± 0.16% for F5), where all the other variables were held constant, and this might be reckoned to its longer chain length.^37^ This is perhaps attributable to the SAA chemical structure; all tweens have the same head group but vary in the length of their respective alkyl chains, as increasing the length of the alkyl chain promotes entrapment efficiency.^38^ Tween 80 has a molecular formula of C_58_H_124_O_26 _(hydrophobic group oleate C_18_, double bond), a molecular weight of 1310 g/mol, and an HLB of 15.0. That’s why it has a longer alkyl chain than T_20_, which possesses a molecular formula of C_58_H_114_O_26_ (hydrophobic group Laurate C_12_), a molecular weight of 1228 g/mol, and an HLB of 16.7. Consequently, it diminishes membrane permeability and therefore promotes drug encapsulation efficiency.

 These findings were consistent with those of Dharashivkar et al,^39^ who revealed that the length of the alkyl chain is an important determinant in entrapment ability, and hence, longer-chain SAAs had improved entrapment effectiveness. Likewise, the alkyl chain length affects the SAA’s HLB value, which in turn impacts drug EE.^21,40^ The level of EE was improved as the SAA’s lipophilicity increased (HLB value decreased).^21,38^ Similarly, in the scenario of NISNV prepared using T_80_ (HLB 15) and T_20_ (HLB 16.7), T_80_ was superior to T_20_, where the lower the SAA HLB, the greater the drug EE as well as stability.^41^

 Concerning the influence of the SAA/CH ratio (X_2_), it was established that upon increasing the ratio from 1:1 (F1) to 4:1 (F3), the EE% was significantly enhanced *(P* < 0.0001), exceeding 80.42 ± 0.30% for the former and 88.02 ± 0.01 for the latter. When assessing the features and conduct of NISNV bilayers, the SAA:CH ratio is crucial.^42^ Upon increasing the SAA:CH ratio, the concentration of SAA increased and the CH decreased; the proportion 1:1 contained 50% SAA and 50% CH, while 4:1 contained 80% SAA and 20% CH, demonstrating a marked decrease in the EE% of Bac-loaded NISNV. This might be ascribed to the rupture of the vesicles’ normal bilayered structure following the addition of CH at a certain concentration, resulting in decreased entrapment.^43,44^ Furthermore, competition between increased levels of CH and the drug for packing spaces inside the bilayers may result in the drug’s exclusion when amphiphiles assemble into the vesicles.^44^

 In addition, increasing the level of factor X_3_ (sonication time) led to a significant reduction (*P* < 0.0001) in EE% where the NISNV dispersion formulated via 0 min sonication exhibit (F5; 73.22 ± 0.16%) and 3 min sonication exhibit (F6; 60.40 ± 0.28%). This was evident because raising the sonication duration reduces the PS of Bac-loaded NISNV, restricting the interior available space for drug charging and hence lowering the EE%.^45^ These findings are per Al-mahallawi et al,^46^ who stated that extending the sonication duration from 0 to 2 minutes reflected a considerable reduction in the EE% of methotrexate-loaded vesicles.

###  Particle size analysis and zeta potential measurements

 The various Bac NISNV particle size values are cited in Table 2 and their zeta potential outcomes are cited in Table 3. The impact of surfactant type (A), surfactant: cholesterol ratio (B), and sonication time (C) on EE% and the impact of surfactant type (a), surfactant: cholesterol ratio (b), and sonication time (c) on PS are illustrated in Figure 1. The output data of the 2^3^ factorial analyses of Bac formulae is shown in Table 4 and the experiment variables’ restrictions as well as the overall desirability outcomes are shown, respectively, in Tables 4 and 5. Results revealed that all Bac NISNV formulae possess a considerably small size, with mean values ranging from 174.64 ± 0.93 nm (F4) to 1174.50 ± 3.54 nm (F1). All the factors (X_1_, X_2_, and X_3_) displayed a significant difference on PS (Y_2_).

**Table 3:** Characterization test outcomes for Bac-loaded NISNV formulae

**Formula**	**ZP (mV)**	**PDI**	**Time (h)**	**Cumulative Amount of Bac Permeated (µg/cm**^2^**)**	
				**F4 gel**	**Bac gel**
F1	-20.61 ± 0.19	0.830 ± 0.09	1	113.94 ± 2.79	44.02 ± 2.84
F2	-26.60 ± 0.15	0.484 ± 0.02	2	210.33 ± 10.84	58.91 ± 2.76
F3	-15.33 ± 0.11	0.431 ± 0.00	3	361.27 ± 15.04	66.89 ± 4.87
F4	-38.12 ± 0.13	0.300 ± 0.03	4	477.45 ± 15.88	80.02 ± 2.13
F5	-14.77 ± 0.21	0.760 ± 0.01	5	547.82 ± 19.67	108.53 ± 6.03
F6	-11.10 ± 0.27	0.630 ± 0.10	6	630.75 ± 21.75	160.64 ± 7.52
F7	-10.00 ± 0.16	0.490 ± 0.04	7	623.78 ± 10.45	199.00 ± 6.36
F8	-17.83 ± 0.08	0.800 ± 0.11	8	630.09 ± 3.53	210.00 ± 3.53

Outcomes are expressed as the average value ± SD, n = 3. ZP: Zeta Potential; PDI: Poly Dispersity Index.

**Table 4:** Output data of 2^3^ factorial analyses of Bac-loaded NISNV formulae

**Responses**	**R**^2^	**Adjusted R**^2^	**Predicted R**^2^	**Adequate precision**	**Significant factors**
EE (%)	0.92	0.90	0.86	20.83	X_1, _X_2, _X_3_
PS (nm)	0.87	0.84	0.78	14.87	X_1, _X_2, _X_3_
**Response**		**EE (%)**		**PS (nm)**	
Observed values		84.53		174.64	
Predicted values		88.14		169.67	

X_1_: surfactant type; X_2_: surfactant/cholesterol ratio; X_3_: sonication time (min); EE: entrapment efficiency; PS: particle size.

**Table 5:** The implemented constraints for the trial variables in addition to the overall desirability outcomes

**Name**	**Goal**	**Lower Limit**	**Upper Limit**	**Lower Weight**	**Upper Weight**	**Importance**
X1: SAA type	In range	T_80_	T_20_	1	1	-
X2: SAA/CH Ratio	In range	1:1	4:1	1	1	-
X3: Sonication time	In range	0	3	1	1	-
Y_1_: EE (%)	Maximize	60	88.04	1	1	+ + + + +
Y_2_: PS (nm)	Minimize	173.98	1177	1	1	+ + + + +

SAA: surfactant; SAA/CH: surfactant/cholesterol ratio; EE: entrapment efficiency; PS: particle size.

 Concerning the influence of X_1_ (SAA type) on PS, a significant reduction (*P* < 0.0258) in PS was noted upon fluctuating from NISNV prepared with T_80_ to that prepared using T_20_. The chain length of the SAAs utilized may alter the PS of Bac NISNV prepared with Tweens^46,47^; as previously described, SAAs with longer alkyl chains produced larger vesicles.^40^ In general, vesicles formulated using T_80_ were bigger than those with T_20_.^47^ The PS of Bac NISNV reduced considerably in the following manner: (1174.50 ± 3.54 nm) for T_80_ (F1) and (806.99 ± 0.01 nm) for T_20_ (F5), as explained in Table 2. These outcomes were comparable to previously published work,^40,46^ where various Tweens were employed as nonionic SAAs. Furthermore, Bayindir and Yuksel^48^ reported equivalent findings while preparing paclitaxel NISNV for oral drug administration utilizing Tweens.

 Regarding the impact of X_2 _(SAA/CH ratio) on PS, it was observed that upon increasing the ratio from 1:1 to 4:1, an obvious decrease (*P* < 0.0001) in PS was achieved, where the alteration was from (1174.50 ± 3.54 nm) for F1 to (420.30 ± 0.42 nm) for F3. In general, regardless of the kind of SAA used, the diameter of NISNV rises as the quantity of CH in the formulation increases. This is possible because CH is a rigid molecule with an inverted cone shape. When hydrated above the gel/liquid transition point, it intercalates between the fluid hydrocarbon chains of the SAA, expanding the size of the vesicle.^12^

 Furthermore, increasing the sonication period (X_3_) considerably lowered the PS (*P* < 0.0003). Increasing the sonication period resulted in more sonication energy being released into the dispersion medium, resulting in a lower PS, where the PS falls from (420.30 ± 0.42 nm) (F3) to (174.64 ± 0.93 nm) (F4), where the former was prepared with 0 min sonication and the latter was prepared with 3 min sonication.^45^ Parallel findings were published by Al-mahallawi et al,^46^ where raising the sonication time for methotrexate NISNV preparation caused a fall in PS.

 The PDI ranged from 0.300 ± 0.03 to 0.830 ± 0.09, confirming that the preparations were homogeneous. The ZP of Bac-loaded NISNV fluctuated from (-10.00 ± 0.16 mV) to (-38.12 ± 0.13 mV). Negative values originate from the ionization of free hydroxyl groups throughout CH.^49^ These findings reflected prior studies that found negative ZP values in nonionic SAA vesicles.^50,51^ Hashim et al^44^ elucidated similar outcomes, where acitretin-loaded NISNV, derived from spans and CH, exhibited ZP values in the range between (-20.77 ± 0.81 mV) and (-41.20 ± 0.36 mV), revealing that the obtained NISNV possessed just enough charge to prohibit aggregation. Because of the existing electrical repulsion between these particles, ZP values around 30 mV generally characterize stable nanosystems.^44^

###  In-vitro release of baclofen from the differently prepared Bac-NISNV formulae

**Figure 2:**
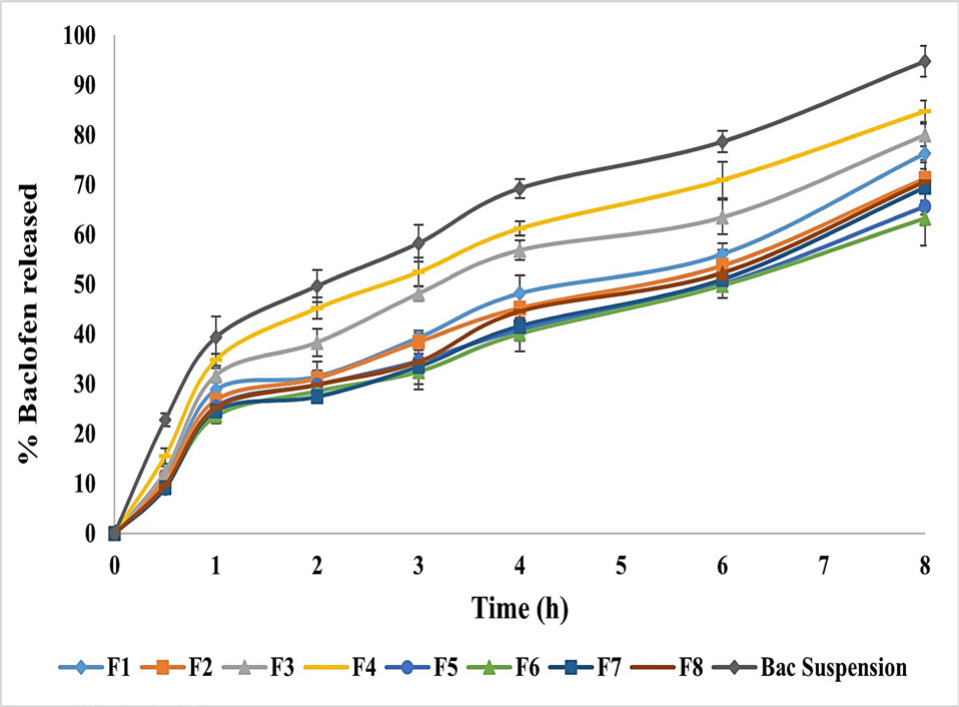
*In-vitro* release of Bac-loaded NISNV formulae against Bac suspension. Outcomes expressed as average value ± S.D (n = 3)

Figure 2 displays the *in-vitro* release profile of all prepared Bac-loaded NISNV, where all formulae were able to release Bac sustainably.^52^ The drug released % after 8 h fluctuated from 63.25 ± 5.47% (F6) to 84.79 ± 3.75% (F4). A significant difference (*P < 0.001*) was detected upon comparing the Bac-loaded NISNV with the pure Bac suspension (94.75 ± 3.14%). The enhanced *in-vitro* release from the Bac-loaded NISNV compared to Bac suspension could be attributed to the nanosize of the formed nanovesicles and the incorporation of the nonionic surfactants that facilitated Bac diffusion from the prepared formulations to the aqueous dissolution medium.^24^

Figure 2 revealed that T_20_ and T_80_ showed a decrease in *in-vitro* drug release in 8 hours compared with Pure Bac suspension. Formulae containing T_20_ showed an average reduction ranging from 10.51% to 19.52%, whereas those containing T_80_ showed a reduction of 25.44% to 33.24%. This might be ascribed to SAA solubilization in vesicles, which resulted in the initial increased drug release.^53^ As displayed in Figure 2, all prepared NISNV formulae possessed biphasic release performance comprised of rapid initial release (28.5-45.22%) of Bac released in the first 2 h, preceded by a delayed second release stage up to 8 hours,^54^ signifying the controlled release of Bac. Similar outcomes were reported by Hassan et al,^55^ where biphasic metformin release from NISNV was observed. The initial fast-release rate is often attributed to drug release from the lipid-soluble component of the NISNV, which contributes to achieving the optimum loading dosage, whereas the subsequent slow-release rate arises from prolonged drug release from the prepared vesicles’ inner lamellae.^21,25^ After 8 h, NISNV comprising T_80_ (F1) as a SAA revealed higher Bac release (76.25 ± 2.14%) than that of T_20_ (F5) (65.68 ± 3.82%), and this enhanced rate of release was attributed to T_80_ unsaturation. This is consistent with the hypothesis that chain unsaturation promotes chain fluidity and permeability.^21^ The fatty acid chain length of polyoxyethylene sorbitan-type surfactants influences drug release. Based on releases from both T_20_ and T_80_, Tween 80 (C9 = 9) > Tween 20 (C12). The greater the chain length, the slower the release rate. When comparing the release of Tween 80 with C9 = 9 and Tween 20 with C12, this is in accordance with the concept that the unsaturation in the chain increases chain fluidity and permeability.^25^ Alterations in vesicle size, lamellarity, and membrane flexibility as a function of SAA chain length and CH concentration may account for differences in *in vitro* release patterns.^21^

###  Evaluation tests of the optimized Bac NISNV formula

####  Ex-vivo permeation of F4 formula through excised newborn rat skin

 As revealed in Table 3, F4 delivered 630.09 ± 3.53 µg/cm^2^ of Bac over 8 h, equates to 100% of Bac. Meanwhile, Bac gel delivered 210.00 ± 3.53 µg/cm^2^ over the same period, which equates to 31.96% of Bac. This means that the amount of Bac permeated from the optimized NISNV gel represents 3 folds of the Bac gel. This might be attributed to NISNV’s capacity to bond with stratum corneum (SC) lipids due to the presence of CH in both the cell membrane and the NISNV structure. This leads to increased Bac concentration at the skin surface and encourages Bac absorption into the deep dermal layers. Tweens in NISNV could also serve as a minor penetration booster.^46,56,57^

 Another scenario for the boosted permeability of the F4 Bac-loaded NISNV formula could be attributed to its ability to disrupt the SC. The use of Bac-loaded NISNV (F4) is the most efficient strategy for improving the permeability of medications due to the entrapment and encapsulation of Bac in NISNV, which cause a decrease in trans-epidermal water loss. This enhances SC hydration and loosens its tightly packed cellular structure, allowing systemic absorption via dermal microcirculation. In addition, NISNV adsorption and/or fusion on the skin’s surface can result in a significant drug thermodynamic activity gradient at the interface, which is the driving factor for drug permeation to the SC.

####  Fourier transform infrared spectroscopy (FT-IR)

**Figure 3:**
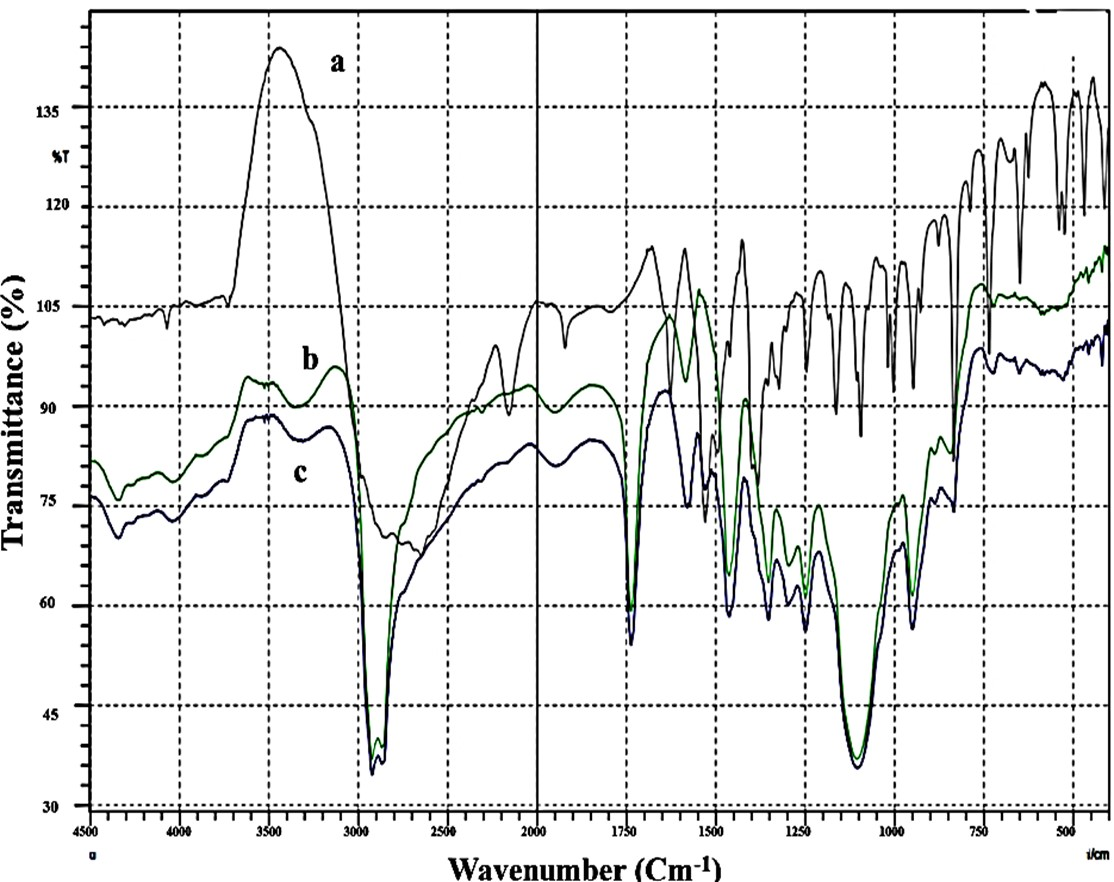
FT-IR charts of a: Bac, b: Plain F4 and c: F4

 The pure Bac powder, the freeze-dried plain F4, and the F4 FT-IR spectra are displayed in Figure 3. The FT-IR spectrum of Bac revealed characteristic peaks at 3150 cm^-1^ attributed to (N-H_2_ stretching), 2920 cm^1^ and 2850.54 cm^-1^ attributed to (aromatic C-H), 2650 cm^-1^ attributed to (-OH group of the acid), 2150 cm^1^ attributed to (alkynyl C≡C stretching), 1925.875 cm^1^ attributed to (C = O stretching), 1625 cm^-1^ attributed to (alkenyl C = C stretching), and 1525 cm^-1^ attributed to (C-C stretch). The peaks that formed at less than 1475 cm^-1^ were thought to be a fingerprint for Bac. (1375 cm^-1^ (O-H bending), 1160 cm^-1^ (C-O stretching), and 830 cm^-1^ (C-Cl stretching)). These findings are consistent with all those outlined in the literature for Bac.^58^ FT-IR spectra of plain F4 and F4 were similarly representative of no interaction that took place between Bac and excipients.^59^

####  X-ray powder diffraction (XRD)

**Figure 4:**
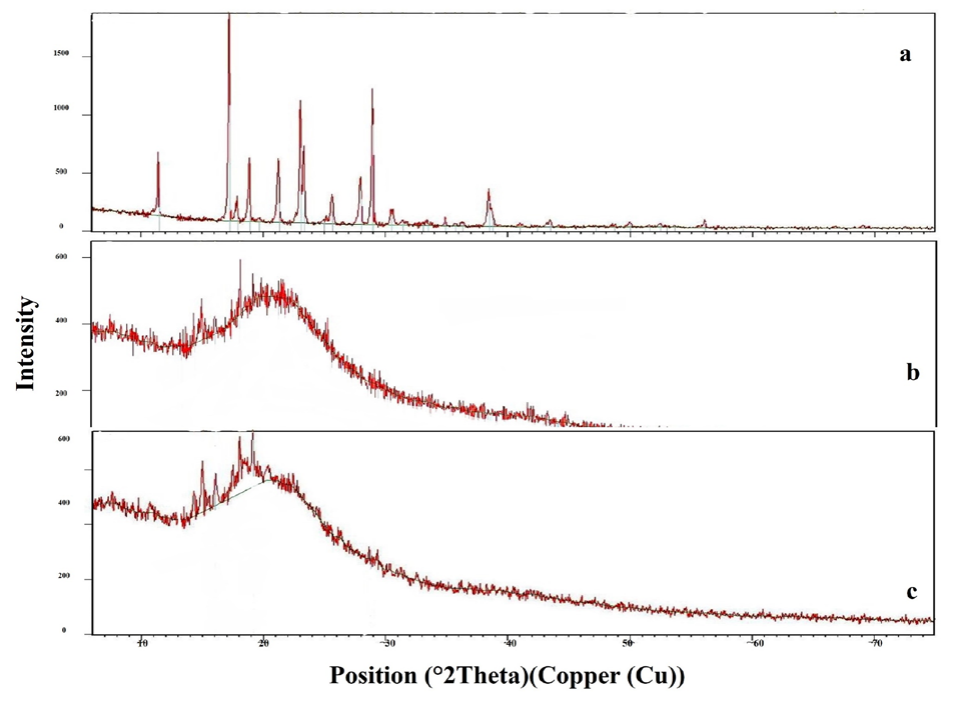
X-Ray diffraction Scans of a: Bac, b: Plain F4 and c: F4

 The X-ray of Figure 4 shows the pure Bac (Figure 4 a), the freeze-dried plain F4 (Figure 4b), and the loaded Bac NISNV F4 (Figure 4c). The XRD pattern of pure Bac displayed strong and pronounced peaks implying crystallinity at a sharp, distinct peak, notably at 2°. The diffraction angles of 11.42°, 17.20°, 18.85°, 19.67°, 21.24°, 23.08°, 25.64°, 26.54°, 27.94°, 28.92°, 30.53°, 31.39°, 33.32°, 34.88°, 35.71°, 36.28°, 38.46°, and 41.00° regarding plain F4 crystallinity were attenuated. In the instance of the F4 diffractogram, a small number of low-intensity signals were observed, which confirmed Bac dispersion within NISNV. These data confirm the concept that the NISNV formula can reduce the crystallinity of pure Bac while maintaining its amorphous character.^60^ As an overall conclusion, F4 showed more diffused peaks indicative of the amorphous form of the entrapped Bac in vesicles.^59,61^ Previously, similar outcomes had been stated for different drugs loaded into NISNV.^62^

####  Surface morphology via transmission electron microscopy (TEM)

**Figure 5:**
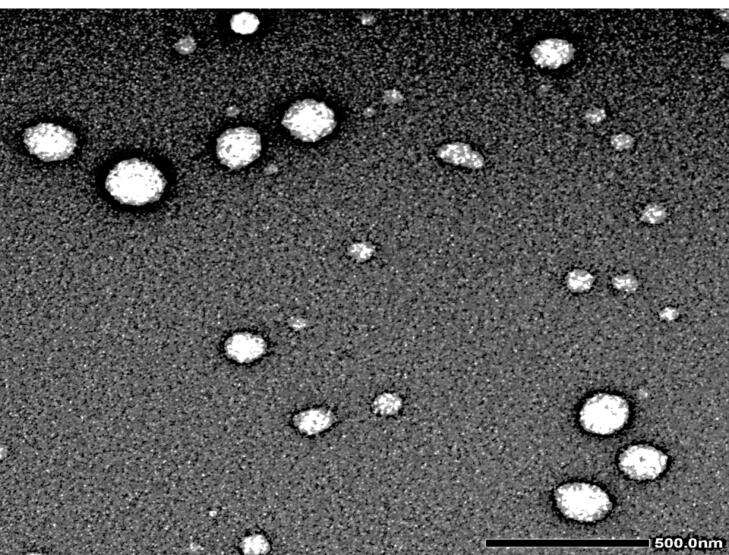
Transmission electron microscopy of F4

 It was revealed in the F4 TEM micrograph that a nano-sized, non-aggregated, well-stained NISNV vesicle structure possessed a smooth surface with a definite wall and core. The vesicles are distinct in their uniform size; F4 has a light-stained internal aqueous space representing the inner hydrophilic domain and a black-stained outer lipophilic domain,^46^ as revealed in Figure 5.

###  Pharmacological investigation outcomes

 The reallocation/repositioning of previously approved drugs is occupying the researcher’s plans for economic demands, safety concerns, and to decrease the time consumed in finding new drugs.^63,64^ And for the same intention, in this work, we have chosen Bac, the specific GABA_B_ receptor agonist, for its known role in neurodegenerative,^65^ and non-neurodegenerative^66^ diseases.

 Chronic traumatic encephalopathy (CTE) is a novel neurodegenerative term that is being introduced nowadays. CTE is thought to be a major consequence of RTBI, as observed in post-mortem contact sports athletes and military members exposed to blows. Hence, it has been linked to repeated concussive and sub-concussive head injuries.^67^ TBI pathogenesis and outcomes are primarily influenced by the biochemical signaling cascades that are triggered by the primary injury and exaggerated by the secondary one. It is feasible that knowledge gained from single or repeated traumas sheds light on the cellular and molecular mechanisms underlying CTE.^68^

###  Selecting the effective dose of oral Bac in the mRTBI model

**Figure 6:**
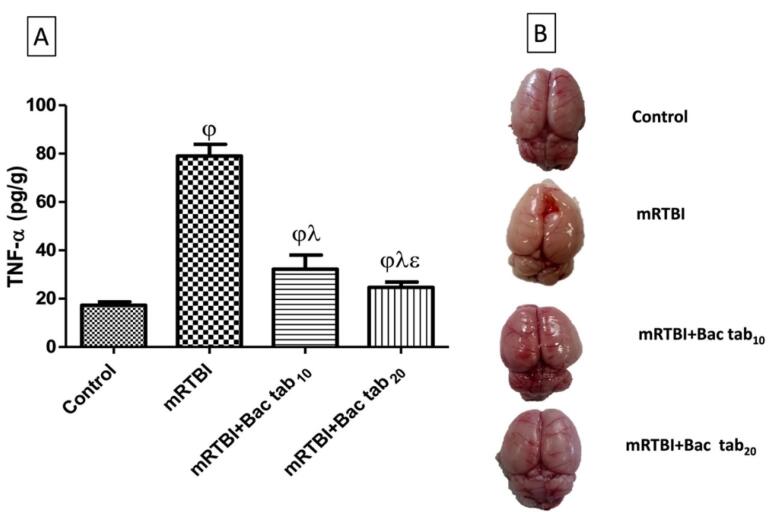
Effect of Bac tab_10 or 20 _mg/kg for seven days on the cerebral cortical contents of (A) TNF-a in rats exposed to mRTBI. (B) Representative images of the cortical contusion area of different groups showing intact brain, normal, preference, and preservation of the morphology after Bac tab_20 _mg/kg more than Bac tab_10 _mg/kg as compared to the untreated traumatized rats’ brain by weight-drop model. As compared with (j) control, (l) mRTBI, and (e) mRTBI + Bac tab_10_ treated groups, n = 8, *P* < 0.05. mRTBI: mild repetitive traumatic brain injury one week after the last hit; Bac: baclofen; and TNF-a: tumor necrosis factor alfa

 As shown in Figure 6, rats exposed to repeated trauma presented a significant cortical increase in (A) TNF-α, a cytokine that triggers inflammation, and (B) the morphological and gross appearance of the whole brain as compared to un-traumatized rats. On the other side, post-treatment with oral Bac (Bac tab_10_: 10 mg/kg and Bac tab_20_: 20 mg/kg) for a week prevented the inflammatory response of trauma and significantly lessened TNF-α cortical contents (*P <*0.05). As compared to the two doses, Bac tab_20 _resulted in a more diminished TNF-α content (*P <*0.05). This effect was extended to morphological appearance, where the post-treatment with Bac tab_20 _revealed an intact brain, normal preference, and preserved morphology as compared to the untreated traumatic rats’ brains. From the previous results, the dose selected to investigate the Bac transdermal formula (F4) effect on mRTBI to achieve the current investigation aim was 20 mg/kg.

###  Identification of the neuroprotective capacity of Bac and screening of the efficacy of F4 against mRTBI

####  Histopathological examination of the right cerebral cortex

**Figure 7:**
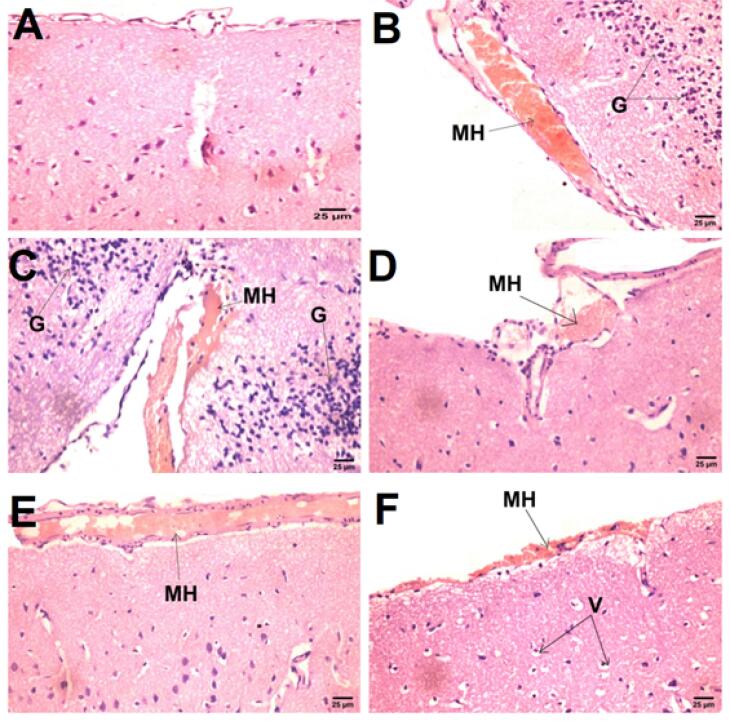
Effect of Bac treatments on histopathological alterations in rats exposed to mRTBI.Photomicrograph showing **(B & C)** meningeal hemorrhage [MH] and diffuse gliosis [G] in the mRTBI group as compared to the normal histological structure of the brain in the **(A) **control negative group. **(D)** mRTBI + Bac tab_20_ presented low meningeal hemorrhage [MH] with vacuolar degeneration in neurons [V], while **(E)** mRTBI + Bac gel didn’t rescue the brain, showing persistent meningeal hemorrhage [MH]. Finally **(F)**, mRTBI + F4 treatment after repeated trauma induction decreased meningeal hemorrhage [MH], showing the highest or chief neuroprotection(Hematoxylin and Eosin stain)

 As displayed in Figure 7, histopathological examination revealed normal brain architecture and structure in the negative control group (A), while a marked increase in meningeal hemorrhage with diffuse gliosis was found in the positive control mRTBI group (B & C). In contrast, the post-treatment with Bac tab_20 _(D) and F4 (F) showed neuroprotection, which was demonstrated by a significant diminution in meningeal hemorrhage. However, the least protective effect was observed in the Bac gel group (E), where the meningeal hemorrhage was significantly apparent when compared to the other treatment groups.

###  Effect of Bac treatments on cerebral cortex gene expression of GABA_B_ receptors, PKC-α, and FAK

**Figure 8:**
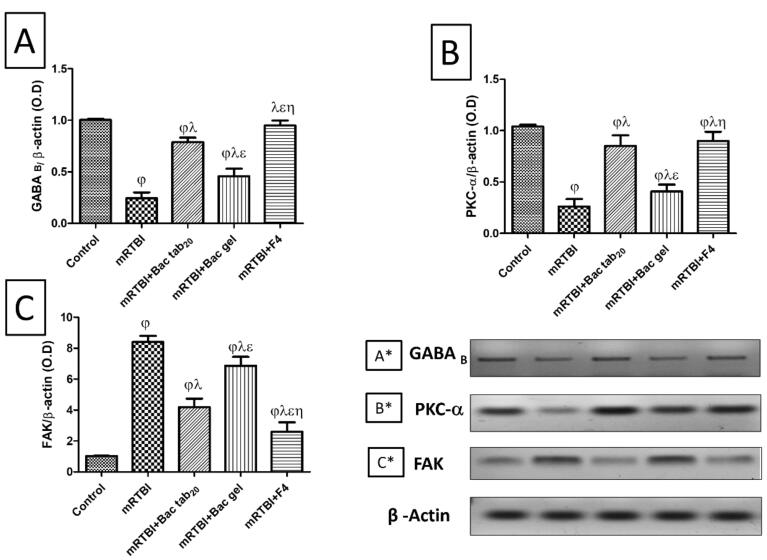
Effect of Bac treatments (for seven days) on cerebral cortical gene expression by western blot analysis of (A, A*) GABA B, (B, B*) PKC-α, and (C, C*) FAK in rats exposed to mRTBI. As compared with (j) control, (l) mRTBI, (e) mRTBI + Bac tab_20_, and (h) mRTBI + Bac gel-treated groups, n = 8, *P* < 0.05. Bac: baclofen; FAK: focal adhesion kinase; GABA_B_: γ-aminobutyric acid B receptors; mRTBI: mild repetitive traumatic brain injury one week after the last hit; and PKC-a: protein kinase C-a

Figure 8 represents the post-induction of mRTBI-associated expression of GABA_B_ receptors (A) and its downstream molecule PKC-α (B), was dramatically reduced (*P* < 0.05), while focal adhesion kinase (FAK) expression (C) was elevated (*P* < 0.05) upon comparison with the negative control group. Contrarily, Bac tab_20, _and F4 reversed all the previous findings relevant to the mRTBI group. Decidedly, the least efficacy among the treatment groups was the Bac gel group at *P* < 0.05.

 Formerly, it was reported that GABA receptor subunits (GABA_A _and GABA_B_) functioned extremely poorly after TBI. Henceforth, Becerra et alstated that the pathophysiology of TBI is decisively influenced by GABAergic dysfunction.^69^ For a well-functioning nervous system, the glutamatergic and GABAergic systems must be balanced. The main excitatory neurotransmitter is glutamate, while the primary inhibitory one is GABA, which is synthesized from glutamate in inhibitory neurons.^70^ Nevertheless, the brain’s excitatory pathways are controlled by GABA, and when GABA-producing cells are lost after injury, the balance between excitation and inhibition is agitated, which causes more cell damage and even neuronal death. GABA is one of the neurotransmitters that may serve as a biomarker of cognitive dysfunction in TBI.^71^ This was confirmed by a previous clinical study that included professional boxers, who presented lower cognitive functions and skills with low prefrontal GABA levels. Therefore, memory impairment and lower cognitive function may be neurochemically correlated with changes in cortical GABA levels.^72^

 In our previous work, we shed light on the glutamate role in RTBI by using MK801, the N-methyl-D-aspartate (NMDA) receptor non-competitive blocker.^33^ On the other side, the GABA role was not fully elucidated in RTBI. Henceforward, we investigated the GABA_B _subunit and its modulation by Bac. We found that all rats exposed to repeated trauma showed a downregulation in GABA_B_ receptors as compared to healthy, un-traumatized rats. However, treatment with Bac tab_20_ and F4 prohibited this effect. Consistent with our findings, an earlier study explained the Bac-neuroprotective ability by modulating GABAergic signaling against 1-methyl-4-phenyl-1,2,3,6-tetrahydropyridine-induced parkinsonism-like manifestations in rats.^35^ Additionally, hippocampal CA1 pyramidal cells exposed to chronic cerebral hypoperfusion were protected by the chronic administration of Bac.^73^

 Over and above that, we investigated the GABA_B_ downstream molecule, PKC-α, which is highly linked to neurodegeneration as a survival molecule in repeated traumas. This was previously documented by Soubh et al,^34^ where increasing its expression enhanced the behavioral outcome and histopathological findings by modulating different signaling molecules that finally prevented hyperphosphorylation tauopathy. In the current study, Bac treatment, either orally (Bac tab_20_) or transdermally (F4), reversed the decrease in PKC-α expression because of repetitive trauma. In the same context, PKC-α was previously shown to be activated by Bac therapy, where a former study stated that Bac improved Fragile X syndrome symptoms through the upregulation of Fragile X mental retardation protein synthesis *via *PKC-dependent signaling.^74^

 Focal adhesions are especially important protein complexes that connect the cytoskeleton with the extracellular matrix via integrins, where integrin interaction activates FAK, starting different mechano-transduction events in almost all cell types. FAK, the non-receptor tyrosine kinase, is essential for a diversity of cellular activities, such as cell migration, proliferation, survival, and control of several signaling pathways.^75^ According to our knowledge, the role of FAK signaling, specifically in repetitive trauma or even CTE, is neither clear nor investigated, despite excessive clinical studies on several solid tumors.^76^

 Our results demonstrated a massive increase in the protein expression of FAK in rats exposed to repeated concussive hits as compared to normal, un-traumatized ones. A previous report constituted that forces directed through different adhesion molecules and their downstream molecule FAK could amplify injury levels that immense the pathological damage and finally accounts for the clinical symptoms seen in diffuse axonal injury.^77,78^ Intriguingly, treatment with Bac tab_20 _or F4 for seven days downregulated FAK expression after mRTBI. Supporting our findings, a former study recorded neuroprotection with FAK inhibitor treatment in female mice with ischemic stroke.^79^ Alongside, the blockage of FAK-mediated signaling pathways may be neuroprotective because of its implications for the cellular mechanism of traumatic axonal injury.^77^ The previously mentioned information increases the perceptivity to agents that can modulate or inhibit FAK and may have high therapeutic value in TBI treatment, where scanty data are available about FAK’s effect on neurological disorders.^79^

###  The effect of Bac treatments on serum TNF-α levels and nuclear factor kappa-B (NF- κB p65) protein expression in immune-stained cortical brain tissue

**Figure 9:**
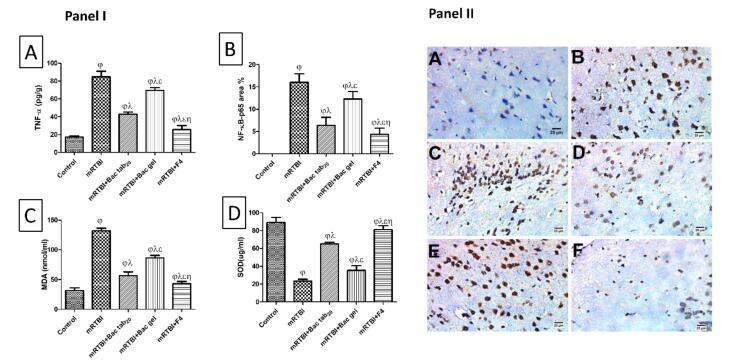
Panel I: effect of Bac treatments for seven days on cerebral cortical contents of **(A)** TNF-α and **(B)** IHC expression of NF-κB p65, as well as serum levels of **(C) **MDA, and **(D)** SOD in rats exposed to mRTBI. Panel II: Photomicrograph showing **(A)** the control negative group presented a negative reaction for NF-κB in the nuclei of neurons, whereas **(B, C)** mRTBI and **(E)** mRTBI + Bac gel demonstrated a severe positive reaction for NF-κB in the nuclei of neurons. Contrarily, **(D)** mRTBI + Bac tab_20_ showed a moderate positive reaction for NF-κB nuclei of neurons, followed by **(F)** mRTBI + F4 transdermal treatment, which presented a mild positive reaction for NF-κB nuclei of neurons (IHC- peroxidase -DAB). As compared with (j) control, (l) mRTBI, (e) mRTBI + Bac tab_20_, and (h) mRTBI + Bac gel-treated groups: n = 8, P < 0.05. Bac: baclofen; MDA: malondialdehyde; mRTBI: mild repetitive traumatic brain injury one week after the last hit; NF-κB p65: Nuclear Factor Kappa-B; TNF-a: tumor necrosis factor alfa; and SOD: superoxide dismutase

 As exhibited in Figure 9 panel I (A), the mRTBI group’s serum TNF-α levels were noticeably higher than the control group (*P* < 0.05). While both groups treated with Bac gel or Bac tab_20 _revealed a notable (*P* < 0.05) decrease in TNF-α in comparison to the mRTBI group, though the group treated with F4 showed the best (*P* < 0.05) comparable ameliorating effect on this parameter compared to other treatment groups. Figure 9, panels I (B) and II, displayed a marked (*P* < 0.05) concomitant elevation of NF-κB p65 expression in immunostained cortical tissue in the mRTBI group in comparison to the healthy control group. Yet, rats treated with Bac gel or Bac tab_20 _after the five repeated hits significantly decreased (*P* < 0.05) NF-κB p65 expression. Remarkably, the group treated with F4 showed the most notable (*P* < 0.05) decline in NF-κB p65 expression compared to other treated groups.

 The inflammatory cascade, according to our previous studies in this milieu, is highly exaggerated and has a significant role in the pathophysiology of both single and repeated TBI.^33,34^ Hence, for the reproducibility of this model and the importance of this process, TNF-α and the parent transcription factor NF-κB p65 were measured. Bac’s different delivery forms, especially F4, demonstrated anti-inflammatory power by halting the increased TNF- levels and decreasing protein expression of NF-κB p65 in immunostained cortical tissues, offering neuroprotection. The anti-inflammatory activity of Bac is clear and well-documented in several animals^80,81^ with clinical studies^82^ supporting our findings.

###  The impact of Bac treatments on serum malondialdehyde (MDA) level and superoxide dismutase (SOD) activity

 As presented in Figure 9, an obvious increase (*P* < 0.05) in serum MDA levels (panel I (C)) with a decrease (*P* < 0.05) in serum SOD activity (panel I (D)) was found in the mRTBI group in comparison to the negative control. Treatment with either Bac gel or Bac tab_20_ significantly restored SOD activity and halted increased MDA levels. While the mRTBI group treated with F4 showed the best (*P* < 0.05) antioxidant potential when compared to other treatment groups.

 As earlier literature mentioned, the previously revealed inflammatory cascades excuse an imbalance between oxidative markers and various antioxidant systems, which leads to oxidative stress that adversely affects normal neuronal function and causes degenerative sequelae.^83^ Oxidative stress increases the lipid peroxidation process and results in many biproducts, such as MDA, a toxic biological marker of oxidative stress. Moreover, the antioxidant defense system is depleted and overwhelmed in response to high oxidative states, such as SOD enzyme.^84^ In the current study, rats exposed to RTBI consumed a high oxidative state, as indicated by the increased levels of MDA and decreased levels of SOD. While Bac treatment balanced the oxidative state, rats treated with Bac tab_20_ or F4 decreased MDA and increased SOD levels as compared to traumatized, untreated rats. In contrast, the stimulation of the GABA_B_ receptor reversed the oxidative stress-damaging consequence by decreasing MDA and replenishing SOD reservoir in AD rat model.^85^ Furthermore, this antioxidant power of Bac observed herein may be the reason behind the decrease in FAK protein expression, where FAK operates as a downstream of both integrin and ROS to enhance the expression of different genes. Besides, it was previously reported that using an antioxidant or ROS inhibitor, such as N-acetyl cysteine, blocks FAK and excessive adhesive signaling in fibrotic diseases.^86^ The anti-inflammatory power of BAC maybe a consequence of its ability to rehabilitate the oxidative state, as increased ROS production has been documented change the balance of pro-inflammatory and anti-inflammatory cytokines, aggravating damage and ultimately causing neuronal and brain death.^87^

 Eventually, the optimized NISNV formula (F4) showed a significant boosted effect in the post trauma treatment, which could be attributed to several reasons. First, the presence of Bac at nano-size within the nanovesicles led to increased drug solubility and dissolution rate, compared to the market tablet.^88^ Second, the transdermal administration of the optimized formula resulted in the prevention of Bac metabolism in liver by the first pass effect, increasing the amount of drug reaching the systemic circulation and enhancing the therapeutic effect.^89^ Third, the incorporation of the surfactant (tween 80) in the NISNV formula led to providing elasticity to the wall of the optimized formula and acting as a permeation enhancer, which led to increasing Bac permeability through the skin, increasing the absorbed fraction of Bac, and enhancing its therapeutic effect.^90^

## Conclusion

 The study outcomes establish the potential of the transdermal administration of Baclofen-loaded NISNV, specifically the optimized formula (F4), as a captivating and neuroprotective therapeutic avenue. This innovative approach holds great promise for alleviating the deleterious effects of secondary injuries associated with mRTBI. Based on our findings, considering GABA_B_ receptors low expression following RTBI and the well-documented GABA role in regulating the brain’s excitatory pathways following brain injury, it is therefore considered a target for TBI treatment, single or repeated. This was proved by restoring brain expression of GABA_B_ and its downstream molecules, PKC-α, and decreasing FAK expression. In addition to its anti-inflammatory effect, it has been shown to decrease serum TNF-α and cortical NF-κB p65. Finally, these effects were reflected as an obvious positive result on oxidative stress, as revealed by decreased MDA levels and increased SOD activity. The results ultimately showcased the immense promise of Baclofen-loaded NISNV as a GABA agonist, as it could remove skin-to-brain barriers. This makes it a good candidate for future clinical trials on single and repeated traumas.

## Competing Interests

 The authors declare no conflicts of interest.

## Data Availability Statement

 All relevant data are within the manuscript, and any other additional materials are available upon request.

## Ethical Approval

 This study was approved by the Research Ethical Committee of the Faculty of Pharmacy, October 6 University (Giza, Egypt), Number: PRE-Ph-2202014.

## Institutional Review Board Statement

 Not applicable.

## Informed Consent Statement

 Not applicable.

## Appendix 1

Particle size (PS) and zeta potential measurement (ZP):

The vesicle size and polydispersity index (PDI) were measured by the Malvern Zetasizer (MAL 104 4595, Malvern, UK). A sample from each formula was diluted with distilled water before analysis (n = 3) at 25°C. The ZP, whether positive or negative, is an important characteristic in determining the stability of NISNV dispersion since it promises good stability reckoned to the high energy barrier among particles and is influenced by its composition and media type. The ZP is a metric of NISNV surface net charge. In the case of ZP, the instrument analyzes the electrophoretic mobility and surface charge.

Absolute ZP values greater than |8-9 mV| are recommended for the stability of the particles, but when the ZP values are less than or equal to |21 mV|, minimal or no agglomeration occurs, whereas |30 mV| is required for full electrostatic stabilization. In the case of low repulsive forces, agglomeration occurs, accompanied by uneven particle distribution and fast settling, indicating an unstable system.

## Appendix 2

In-vitro release of Bac-loaded NISNV

Dialysis tubing (HIMEDIA, cut-off 12,000-14,000 MW) is a commonly used approach for studying in- vitro release, where it performs as a” donor compartment” to be used for the evaluation of in-vitro release studies of Bac-loaded NISNV since it can retain NISNV dispersion and allowing the free drug to migrate into the dissolving liquid. Before the study, the dialysis bag was pre-soaked in distilled water for 10 minutes. A sample from the NISNV dispersion containing 2 mg Bac was pipetted into one portion of the bag, which was then tightened with an additional closure clip to avoid leaking before being situated in a vessel comprising 100 ml of phosphate buffer (PB) (pH 7.4). The vessel was set over a magnetic stirrer at 50 rpm, and the temperature was kept constant at 37°C ± 0.5°C. Aliquots of 5 ml were revoked at 0.5, 1, 2, 3, 4, 6 and 8 h, and hence were replaced with an equal volume of fresh PB medium to maintain a constant medium volume. Bac concentration was determined via UV spectrophotometer absorbance at 267 nm, where a sample from Bac suspension containing 2 mg Bac was taken to be compared with. The test was performed in triplicate, and the mean percentage released was calculated.

## Appendix 3

Fourier transform infrared spectroscopy (FT-IR) and X-Ray powder diffraction (XRD):

Before carrying out FT-IR or XRD, the plain F4 and Bac F4 NISNV dispersions were subjected to lyophilization via lyophilizer (Christ Alpha 1-2LD plus, Munich, Germany) with a condenser at -55 °C and vacuum at 7.6 Pa/24 h for complete dryness with a primary drying cycle at -30 °C and 0.37 mbar/12 h, followed by secondary drying at 20 °C and 0.01 mbar/12 h. The FT-IR spectrum of Bac, plain F4, and Bac F4 was done by mixing each sample with KBr (IR grade) in the ratio of 2:200 (IRAffinity-1, Shimadzu, Japan) at a frequency of 4500-500 cm-1.

The peak position (angle of diffraction) indicates the nature of the sample, either crystalline or amorphous. XRD patterns were determined for Bac, plain F4, and Bac F4. The samples were exposed to Cu-K radiation (45 kV, 40 mA) at a scan rate of 2°/min. The results were then displayed as peak height (intensity) versus 2Ɵ (Advanced Direction system, Scinta g Inc., USA).

## Appendix 4

Collection of samples:

Rats were anesthetized, and blood was collected via retro-orbital bleeding, transferred to plain blood collection tubes, and centrifuged at 3000 rpm for 20 min. Following blood collection, anesthetized rats were decapitated; the whole brain was kept in buffered formaldehyde (10%) for histological examination, while the right cerebral cortex of the brain was dissected for different biochemical analysis.

## Appendix 5

Biochemical measurements:

Serum tumor necrosis factor-α (TNF-α) was assayed by an ELISA kit according to the manufacturer’s instructions (Cusabio Biotech Co., Wuhan, China, Cat. # CSB-E11897r). Also, serum levels of malondialdehyde (MDA) and superoxide dismutase (SOD) were measured using commercial Biodiagnostic kits (Egypt) (Cat. # MD 25 29 and Cat. # SD 25 21, respectively). Adhering to the previously described method28, the radioimmunoprecipitation assay buffer (RIPA) fractions of the cortical homogenates were employed for the Western blot analysis. Thermo Scientific Co. (Waltham, MA, USA) supplied the primary rabbit polyclonal antibodies for gamma-aminobutyric acid (GABAB; 1:500), focal adhesion kinase (FAK; 1:500), and protein kinase C-α (PKC-α; 1:1000). Using Image Lab software version 5.1 and a Chem-iDoc imaging device, specific signals were created (Bio-Rad Laboratories Inc., Hercules, CA, USA). After normalization to β-actin, the data were represented in arbitrary units.

## Appendix 6

Histopathological investigations:

Samples were sectioned into 4 µm-thick sagittal brain slices from the right cerebral hemispheres using a rotatory microtome after being embedded in paraffin wax for histopathological analysis. Using the Leica application module for tissue section analysis, samples were cut, dehydrated in ethanol, cleaned in xylene, and stained with hematoxylin and eosin (H&E) (Leica Microsystems GmbH, Wetzlar, Germany).

For immunohistochemistry, 5 µm slices were sliced into positively charged slides, rehydrated, and exposed to a heat retrieval step before being incubated with primary anti-Nuclear Factor Kappa-B (anti-NF-κB) p65 (at a dilution of 1:100; CAT. #E-AB-32232, Elabscience Biotechnology HO, TX, USA). After being washed, tissue sections were blocked for endogenous peroxidases and treated with a secondary antibody that had been horseradish peroxidase (HRP)-labeled (1:1000). The color was created using a 3,3’- diaminobenzidine tetrahydrochloride (DAB) substrate kit. Slides with negative controls were produced by skipping the primary antibody stage. The Olympus DP-27 camera was used to take pictures, while the Olympus BX43 microscope was used to examine the slides. Using Cell Sens dimensions, positive immune staining was measured as an area percentage (Olympus software).

## References

[1] Aungst SL, Kabadi SV, Thompson SM, Stoica BA, Faden AI (2014). Repeated mild traumatic brain injury causes chronic neuroinflammation, changes in hippocampal synaptic plasticity, and associated cognitive deficits. J Cereb Blood Flow Metab.

[2] Dewan MC, Rattani A, Gupta S, Baticulon RE, Hung YC, Punchak M (2018). Estimating the global incidence of traumatic brain injury. J Neurosurg.

[3] Dixon KJ (2017). Pathophysiology of traumatic brain injury. Phys Med Rehabil Clin N Am.

[4] McCrea MA, Nelson LD, Guskiewicz K (2017). Diagnosis and management of acute concussion. Phys Med Rehabil Clin N Am.

[5] Scorza KA, Cole W (2019). Current concepts in concussion: initial evaluation and management. Am Fam Physician.

[6] Chen L, Chan SC, Yung WH (2002). Rotational behavior and electrophysiological effects induced by GABA(B) receptor activation in rat globus pallidus. Neuroscience.

[7] Kaneda K, Kita H (2005). Synaptically released GABA activates both pre- and postsynaptic GABA(B) receptors in the rat globus pallidus. J Neurophysiol.

[8] Pérez-Arredondo A, Cázares-Ramírez E, Carrillo-Mora P, Martínez-Vargas M, Cárdenas-Rodríguez N, Coballase-Urrutia E (2016). Baclofen in the therapeutic of sequele of traumatic brain injury: spasticity. Clin Neuropharmacol.

[9] Romito JW, Turner ER, Rosener JA, Coldiron L, Udipi A, Nohrn L (2021). Baclofen therapeutics, toxicity, and withdrawal: a narrative review. SAGE Open Med.

[10] Nyirjesy P, Lev-Sagie A, Mathew L, Culhane JF (2009). Topical amitriptyline-baclofen cream for the treatment of provoked vestibulodynia. J Low Genit Tract Dis.

[11] Parmar A, Brijesh S (2018). Niosomes as transdermal drug delivery system. Biomed Res J.

[12] Abdelkader H, Farghaly U, Moharram H (2014). Effects of surfactant type and cholesterol level on niosomes physical properties and in vivo ocular performance using timolol maleate as a model drug. J Pharm Investig.

[13] Ramesh YV, Jawahar N, Jakki SL (2013). Proniosomes: a novel nano vesicular transdermal drug delivery. J Pharm Sci Res.

[14] Bhardwaj P, Tripathi P, Gupta R, Pandey S (2020). Niosomes: a review on niosomal research in the last decade. J Drug Deliv Sci Technol.

[15] Makeshwar KB, Wasankar SR (2013). Niosome: a novel drug delivery system. Asian J Pharm Res.

[16] Carafa M, Santucci E, Lucania G (2002). Lidocaine-loaded non-ionic surfactant vesicles: characterization and in vitro permeation studies. Int J Pharm.

[17] Elshafeey AH, El-Dahmy RM (2021). Formulation and development of oral fast-dissolving films loaded with nanosuspension to augment paroxetine bioavailability: in vitro characterization, ex vivo permeation, and pharmacokinetic evaluation in healthy human volunteers. Pharmaceutics.

[18] Ali AA, Hassan AH, Eissa EM, Aboud HM (2021). Response surface optimization of ultra-elastic nanovesicles loaded with deflazacort tailored for transdermal delivery: accentuated bioavailability and anti-inflammatory efficacy. Int J Nanomedicine.

[19] Elsayed I, El-Dahmy RM, El-Emam SZ, Elshafeey AH, El Gawad NA, El-Gazayerly ON (2020). Response surface optimization of biocompatible elastic nanovesicles loaded with rosuvastatin calcium: enhanced bioavailability and anticancer efficacy. Drug DelivTransl Res.

[20] Fayez SM, Elnahas OS, Fayez AM, El-Mancy SS (2023). Coconut oil based self-nano emulsifying delivery systems mitigate ulcerogenic NSAIDs side effect and enhance drug dissolution: formula optimization, in-vitro, and in-vivo assessments. Int J Pharm.

[21] Nosrati H, Salehiabar M, Davaran S, Danafar H, Kheiri Manjili H (2018). Methotrexate-conjugated L-lysine coated iron oxide magnetic nanoparticles for inhibition of MCF-7 breast cancer cells. Drug Dev Ind Pharm.

[22] Villalobos-Hernández JR, Müller-Goymann CC (2005). Novel nanoparticulate carrier system based on carnauba wax and decyl oleate for the dispersion of inorganic sunscreens in aqueous media. Eur J Pharm Biopharm.

[23] Kakar R, Rao R, Goswami A, Nanda S, Saroha K (2010). Proniosomes: an emerging vesicular system in drug delivery and cosmetics. Pharm Lett.

[24] Patel P, Barot T, Kulkarni P (2020). Formulation, characterization and in-vitro and in-vivo evaluation of capecitabine loaded niosomes. Curr Drug Deliv.

[25] Ruckmani K, Sankar V (2010). Formulation and optimization of Zidovudine niosomes. AAPS PharmSciTech.

[26] Abuelella KE, Abd-Allah H, Soliman SM, Abdel-Mottaleb MM (2023). Skin targeting by chitosan/hyaluronate hybrid nanoparticles for the management of irritant contact dermatitis: in vivo therapeutic efficiency in mouse-ear dermatitis model. Int J Biol Macromol.

[27] Hassen Elshafeey A, Moataz El-Dahmy R (2022). A novel oral medicated jelly for enhancement of etilefrine hydrochloride bioavailability: in vitro characterization and pharmacokinetic evaluation in healthy human volunteers. Saudi Pharm J.

[28] Farghaly DA, Aboelwafa AA, Hamza MY, Mohamed MI (2018). Microemulsion for topical delivery of fenoprofen calcium: in vitro and in vivo evaluation. J Liposome Res.

[29] Moutasim MY, ElMeshad AN, El-Nabarawi MA (2017). A pharmaceutical study on lornoxicam fast disintegrating tablets: formulation and in vitro and in vivo evaluation. Drug DelivTransl Res.

[30] Hu Lt, Bentler PM (1999). Cutoff criteria for fit indexes in covariance structure analysis: conventional criteria versus new alternatives. Struct Equ Modeling.

[31] Sheta NM, Elfeky YA, Boshra SA (2020). Cardioprotective efficacy of silymarin liquisolid in isoproterenol prompted myocardial infarction in rats. AAPS PharmSciTech.

[32] Weber B, Lackner I, Haffner-Luntzer M, Palmer A, Pressmar J, Scharffetter-Kochanek K (2019). Modeling trauma in rats: similarities to humans and potential pitfalls to consider. J Transl Med.

[33] El-Gazar AA, Soubh AA, Mohamed EA, Awad AS, El-Abhar HS (2019). Morin post-treatment confers neuroprotection in a novel rat model of mild repetitive traumatic brain injury by targeting dementia markers, APOE, autophagy and Wnt/β-catenin signaling pathway. Brain Res.

[34] Soubh AA, El-Gazar AA, Mohamed EA, Awad AS, El-Abhar HS (2021). Further insights for the role of Morin in mRTBI: implication of non-canonical Wnt/PKC-α and JAK-2/STAT-3 signaling pathways. Int Immunopharmacol.

[35] Tyagi RK, Bisht R, Pant J, Kumar P, Abdul Majeed AB, Prakash A (2015). Possible role of GABA-B receptor modulation in MPTP induced Parkinson’s disease in rats. Exp ToxicolPathol.

[36] Abd El-Aal SA, Abd Elrahman M, Reda AM, Afify H, Ragab GM, El-Gazar AA (2022). Galangin mitigates DOX-induced cognitive impairment in rats: implication of NOX-1/Nrf-2/HMGB1/TLR4 and TNF-α/MAPKs/RIPK/MLKL/BDNF. Neurotoxicology.

[37] Kayser O, Lemke A, Hernández-Trejo N (2005). The impact of nanobiotechnology on the development of new drug delivery systems. Curr Pharm Biotechnol.

[38] Zaki RM, Ali AA, El Menshawe SF, Bary AA (2014). Formulation and in vitro evaluation of diacerein loaded niosomes. Int J Pharm Pharm Sci.

[39] Dharashivkar SS, Sahasrabuddhe SH, Saoji AN (2015). Niosomally encapsulated silver sulfadiazine gel for burn treatment. J Microencapsul.

[40] Abdelbary G, El-Gendy N (2008). Niosome-encapsulated gentamicin for ophthalmic controlled delivery. AAPS PharmSciTech.

[41] Singh CH, Jain CP, Kumar BN (2011). Formulation, characterization, stability and invitro evaluation of nimesulide niosomes. Pharmacophore.

[42] Zabihollahi R, Motevaseli E, Sadat SM, Azizi-Saraji AR, Asaadi-Dalaie S, Modarressi MH (2012). Inhibition of HIV and HSV infection by vaginal lactobacilli in vitro and in vivo. Daru.

[43] Sjöholm E, Sandler N (2019). Additive manufacturing of personalized orodispersible warfarin films. Int J Pharm.

[44] Abu Hashim II, Abo El-Magd NF, El-Sheakh AR, Hamed MF, Abd El-Gawad AE (2018). Pivotal role of Acitretin nanovesicular gel for effective treatment of psoriasis: ex vivo-in vivo evaluation study. Int J Nanomedicine.

[45] Song X, Zhao Y, Wu W, Bi Y, Cai Z, Chen Q (2008). PLGA nanoparticles simultaneously loaded with vincristine sulfate and verapamil hydrochloride: systematic study of particle size and drug entrapment efficiency. Int J Pharm.

[46] Al-Mahallawi AM, Fares AR, Abd-Elsalam WH (2019). Enhanced permeation of methotrexate via loading into ultra-permeable niosomal vesicles: fabrication, statistical optimization, ex vivo studies, and in vivo skin deposition and tolerability. AAPS PharmSciTech.

[47] Agarwal R, Katare OP, Vyas SP (2001). Preparation and in vitro evaluation of liposomal/niosomal delivery systems for antipsoriatic drug dithranol. Int J Pharm.

[48] Bayindir ZS, Yuksel N (2010). Characterization of niosomes prepared with various nonionic surfactants for paclitaxel oral delivery. J Pharm Sci.

[49] Mohamed LK, Abdelmottaleb M, Geneidi AS (2021). Formulation and characterization of proniosomal gels loaded with levofloxacin for dermal drug delivery. Arch Pharm Sci Ain Shams Univ.

[50] Junyaprasert VB, Teeranachaideekul V, Supaperm T (2008). Effect of charged and non-ionic membrane additives on physicochemical properties and stability of niosomes. AAPS PharmSciTech.

[51] Manosroi A, Khanrin P, Lohcharoenkal W, Werner RG, Götz F, Manosroi W (2010). Transdermal absorption enhancement through rat skin of gallidermin loaded in niosomes. Int J Pharm.

[52] Nandini PT, Doijad RC, Shivakumar HN, Dandagi PM (2015). Formulation and evaluation of gemcitabine-loaded solid lipid nanoparticles. Drug Deliv.

[53] Rasul A, Imran Khan M, Ur Rehman M, Abbas G, Aslam N, Ahmad S (2020). In vitro characterization and release studies of combined nonionic surfactant-based vesicles for the prolonged delivery of an immunosuppressant model drug. Int J Nanomedicine.

[54] Khalil RM, Abdelbary GA, Basha M, Awad GE, El-Hashemy HA (2017). Enhancement of lomefloxacin HCl ocular efficacy via niosomal encapsulation: in vitro characterization and in vivo evaluation. J Liposome Res.

[55] Hasan AA, Madkor H, Wageh S (2013). Formulation and evaluation of metformin hydrochloride-loaded niosomes as controlled release drug delivery system. Drug Deliv.

[56] Aboelwafa AA, El-Setouhy DA, Elmeshad AN (2010). Comparative study on the effects of some polyoxyethylene alkyl ether and sorbitan fatty acid ester surfactants on the performance of transdermal carvedilol proniosomal gel using experimental design. AAPS PharmSciTech.

[57] El-Laithy HM, Shoukry O, Mahran LG (2011). Novel sugar esters proniosomes for transdermal delivery of vinpocetine: preclinical and clinical studies. Eur J Pharm Biopharm.

[58] Mohamed Ali MA, Sabati AM, Abduh Ali B (2017). Formulation and evaluation of baclofen mucoadhesive buccal films. FABAD J Pharm Sci.

[59] Vickers NJ (2017). Animal communication: when i’m calling you, will you answer too?. Curr Biol.

[60] Schäfer AM, Meyer Zu Schwabedissen HE, Grube M (2021). Expression and Function of organic anion transporting polypeptides in the human brain: physiological and pharmacological implications. Pharmaceutics.

[61] Farmoudeh A, Akbari J, Saeedi M, Ghasemi M, Asemi N, Nokhodchi A (2020). Methylene blue-loaded niosome: preparation, physicochemical characterization, and in vivo wound healing assessment. Drug DelivTransl Res.

[62] Gurrapu A, Jukanti R, Bobbala SR, Kanuganti S, Jeevana JB (2012). Improved oral delivery of valsartan from maltodextrin based proniosome powders. Adv Powder Technol.

[63] Türkeş C, Arslan M, Demir Y, Çoçaj L, Rifati Nixha A, Beydemir Ş (2019). Synthesis, biological evaluation and in silico studies of novel N-substituted phthalazine sulfonamide compounds as potent carbonic anhydrase and acetylcholinesterase inhibitors. Bioorg Chem.

[64] Rampa A, Gobbi S, Belluti F, Bisi A (2021). Tackling Alzheimer’s disease with existing drugs: a promising strategy for bypassing obstacles. Curr Med Chem.

[65] Uto-Konomi A, McKibben B, Wirtz J, Sato Y, Takano A, Nanki T (2013). CXCR7 agonists inhibit the function of CXCL12 by down-regulation of CXCR4. BiochemBiophys Res Commun.

[66] Jin S, Merchant ML, Ritzenthaler JD, McLeish KR, Lederer ED, Torres-Gonzalez E (2015). Baclofen, a GABABR agonist, ameliorates immune-complex mediated acute lung injury by modulating pro-inflammatory mediators. PLoS One.

[67] Kulbe JR, Hall ED (2017). Chronic traumatic encephalopathy-integration of canonical traumatic brain injury secondary injury mechanisms with tau pathology. Prog Neurobiol.

[68] Blennow K, Brody DL, Kochanek PM, Levin H, McKee A, Ribbers GM (2016). Traumatic brain injuries. Nat Rev Dis Primers.

[69] Parga Becerra A, Logsdon AF, Banks WA, Ransom CB. Traumatic brain injury broadly affects GABAergic signaling in dentate gyrus granule cells. eNeuro 2021;8(3):ENEURO.0055-20.2021. 10.1523/eneuro.0055-20.2021. PMC8116114 33514602

[70] Guerriero RM, Giza CC, Rotenberg A (2015). Glutamate and GABA imbalance following traumatic brain injury. Curr Neurol Neurosci Rep.

[71] Sun ZL, Feng DF (2014). Biomarkers of cognitive dysfunction in traumatic brain injury. J Neural Transm (Vienna).

[72] Kim GH, Kang I, Jeong H, Park S, Hong H, Kim J (2019). Low prefrontal GABA levels are associated with poor cognitive functions in professional boxers. Front Hum Neurosci.

[73] Liu L, Li CJ, Lu Y, Zong XG, Luo C, Sun J (2015). Baclofen mediates neuroprotection on hippocampal CA1 pyramidal cells through the regulation of autophagy under chronic cerebral hypoperfusion. Sci Rep.

[74] Zhang W, Xu C, Tu H, Wang Y, Sun Q, Hu P (2015). GABAB receptor upregulates fragile X mental retardation protein expression in neurons. Sci Rep.

[75] Lu Y, Sun H (2020). Progress in the development of small molecular inhibitors of focal adhesion kinase (FAK). J Med Chem.

[76] Zhao X, Guan JL (2011). Focal adhesion kinase and its signaling pathways in cell migration and angiogenesis. Adv Drug Deliv Rev.

[77] Hemphill MA. A Role for Focal Adhesions and Extracellular Matrix in Traumatic Axonal Injury [dissertation]. Harvard University; 2014.

[78] Shishido H, Ueno M, Sato K, Matsumura M, Toyota Y, Kirino Y (2019). Traumatic brain injury by weight-drop method causes transient amyloid-β deposition and acute cognitive deficits in mice. Behav Neurol.

[79] Jia C, Lovins C, Malone HM, Keasey MP, Hagg T (2022). Female-specific neuroprotection after ischemic stroke by vitronectin-focal adhesion kinase inhibition. J Cereb Blood Flow Metab.

[80] Duthey B, Hübner A, Diehl S, Boehncke S, Pfeffer J, Boehncke WH (2010). Anti-inflammatory effects of the GABA(B) receptor agonist baclofen in allergic contact dermatitis. Exp Dermatol.

[81] Crowley T, Fitzpatrick JM, Kuijper T, Cryan JF, O’Toole O, O’Leary OF (2015). Modulation of TLR3/TLR4 inflammatory signaling by the GABAB receptor agonist baclofen in glia and immune cells: relevance to therapeutic effects in multiple sclerosis. Front Cell Neurosci.

[82] Friedman BW, Irizarry E, Solorzano C, Zias E, Pearlman S, Wollowitz A (2019). A randomized, placebo-controlled trial of ibuprofen plus metaxalone, tizanidine, or baclofen for acute low back pain. Ann Emerg Med.

[83] Rodríguez-Rodríguez A, Egea-Guerrero JJ, Murillo-Cabezas F, Carrillo-Vico A (2014). Oxidative stress in traumatic brain injury. Curr Med Chem.

[84] Birben E, Sahiner UM, Sackesen C, Erzurum S, Kalayci O (2012). Oxidative stress and antioxidant defense. World Allergy Organ J.

[85] Sun Z, Sun L, Tu L (2020). GABAB receptor-mediated PI3K/Akt signaling pathway alleviates oxidative stress and neuronal cell injury in a rat model of Alzheimer’s disease. J Alzheimers Dis.

[86] Shi-Wen X, Thompson K, Khan K, Liu S, Murphy-Marshman H, Baron M (2012). Focal adhesion kinase and reactive oxygen species contribute to the persistent fibrotic phenotype of lesional scleroderma fibroblasts. Rheumatology (Oxford).

[87] Wu L, Xiong X, Wu X, Ye Y, Jian Z, Zhi Z (2020). Targeting oxidative stress and inflammation to prevent ischemia-reperfusion injury. Front Mol Neurosci.

[88] Shamma RN, Elsayed I (2013). Transfersomal lyophilized gel of buspirone HCl: formulation, evaluation and statistical optimization. J Liposome Res.

[89] Cleary GW. Transdermal controlled release systems. In: Medical Applications of Controlled Release. CRC Press; 2019. p. 203-52.

[90] Som I, Bhatia K, Yasir M (2012). Status of surfactants as penetration enhancers in transdermal drug delivery. J Pharm Bioallied Sci.

